# Understanding the implementation of kidney supportive care interventions to improve palliative care outcomes for adults receiving haemodialysis: protocol for a rapid realist review

**DOI:** 10.1136/bmjopen-2025-114188

**Published:** 2026-07-21

**Authors:** Chalani Lasanthika, Barnaby Hole, Ala Szczepura, Ann Bonner, Jane Coad, Emma Murphy

**Affiliations:** 1 Centre for Healthcare & Community Transformation, Coventry University, Coventry, UK; 2 Department of Nursing & Midwifery, Faculty of Allied Health Sciences, University of Sri Jayewardenepura, Nugegoda, Sri Lanka; 3 Centre for Care Excellence, Coventry University and University Hospitals Coventry and Warwickshire NHS Trust, Coventry, UK; 4 Institute for Cardio-Metabolic Medicine, University Hospitals Coventry and Warwickshire NHS Trust, Coventry, UK; 5 Palliative and End of Life Care Research Group & Population Health Sciences Research Group, University of Bristol, Bristol, UK; 6 North Bristol NHS Trust, Bristol, UK; 7 School of Nursing and Midwifery, Griffith University, Southport, Queensland, Australia; 8 Kidney Health Service, Metro North Hospital and Health Service, Herston, Queensland, Australia; 9 School of Health Sciences, University of Nottingham, Nottingham, UK

**Keywords:** Dialysis, End stage renal failure, Adult nephrology, Adult palliative care, Review

## Abstract

**Abstract:**

**Introduction:**

Chronic kidney disease (CKD) is a significant global health concern, contributing substantially to mortality; in advanced stages, adults often require kidney supportive care (KSC), a model of care/intervention that addresses complex palliative and supportive needs. These needs often remain insufficiently addressed, particularly among adults receiving haemodialysis, due to the interplay of multiple contextual factors and inconsistent reported outcomes. Consequently, it remains unclear how, for whom and under what circumstances KSC works (or does not work). Therefore, this rapid realist review aims to identify the key contexts in which KSC interventions have been implemented and the mechanisms that influenced palliative care outcomes for adults receiving haemodialysis.

**Methods and analysis:**

Rapid realist review is a structured, theory-driven approach to evidence synthesis conducted within a condensed timeframe, aiming to explain how and why interventions work, for whom and in what contexts, to identify effective models of service delivery. This rapid realist review will be conducted following a multiple-step process, including defining the project scope and research questions development (including how the findings will be utilised); development of search terms and identification of relevant literature; quality appraisal; data extraction and preliminary programme theory development; validation and refinement of programme theory with content experts; and dissemination of findings.

A systematic literature search will be conducted from 2020 to 2025 using the following databases: MEDLINE, CINAHL, PsycINFO, Cochrane Central Register of Controlled Trials, Scopus and Academic Search Complete, with supplementary forward and backward citation tracking. Two reviewers will screen titles and abstracts for relevant research, and the full papers selected will be used to extract data and assess the quality of evidence based on relevance, richness and rigour. Data extraction and synthesis will be conducted iteratively to identify context-mechanism–outcome configurations (CMOCs), which will inform the development and refinement of the programme theory, rather than simply informing palliative care outcomes.

A 10-member expert panel will review, evaluate and reflect on the initial CMOCs in light of their clinical expertise and personal experience. The reporting of findings from this review will be guided by the Realist and Meta-narrative Evidence Syntheses: Evolving Standards (RAMESES). This rapid realist review will support service redesign to better address the unmet palliative care needs of adults receiving haemodialysis and inform policy initiatives and education to deliver context-specific KSC across the UK and globally.

**Ethics and dissemination:**

This study received ethics approval from Coventry University Ethics Committee (Ref-P182536). Results will be disseminated to stakeholder groups and by means of publication in a peer-reviewed journal and presentation at international scientific conferences.

**PROSPERO review registration:**

CRD420251000111.

**STRENGTHS AND LIMITATIONS OF THIS STUDY**

- The rapid realist approach is better suited than other empirical methods to understand the mechanisms behind why and how kidney supportive care (KSC) works or does not work for adults receiving haemodialysis, rather than assuming there is one model to suit all situations.
- Diverse evidence synthesis from multiple sources, including expert panel insights at all stages of the review and an iterative approach to literature search, might potentially influence the robustness of the review, ensuring the real-world applicability of findings.
- A detailed understanding of the mechanisms, defined as the underlying processes, such as participants’ responses to resources or changes in care that lead to successful KSC interventions and the contexts in which these mechanisms are triggered, will support service redesign.
- As the search is limited to the evidence published in the English language, this may restrict generalisability to non-English speaking countries.

## Introduction

 Chronic kidney disease (CKD) is a significant global health problem, which is prevalent among approximately 9.5% of people worldwide. CKD is currently the third-fastest-growing cause of mortality and is expected to be the fifth leading cause of premature mortality by 2040.^1 2^ CKD affects individuals of all ages, yet the prevalence is higher (almost 50%) among those over 70 years,^3^ who experience more advanced stages of the disease.^4^

CKD can be defined as ‘abnormalities of kidney structure or function, present for a minimum of three months, with health implications’.^5^ ‘Kidney failure’ occurs when renal function declines rapidly, with glomerular filtration rate falling below 15 mL/min/1.73 m^2^.^5 6^ Adults with kidney failure are treated with different treatment modalities, including kidney replacement therapy (KRT) such as dialysis (either haemodialysis or peritoneal dialysis), kidney transplantation and conservative kidney management. Haemodialysis is the most prevalent form of KRT globally, representing 69% of all KRT and 89% of all dialysis.^7^

The care for adults with kidney failure may have to address complex needs, particularly in the clinical, psychosocial and spiritual domains and require vigilant and respectful coordination between specialised health services, including routine nephrology care, palliative care and general practice.^8^ The complexity of care of these individuals is often accompanied by numerous supportive care needs requiring palliative care support, referred to as ‘renal palliative care’ or kidney supportive care (KSC).^9^ KSC focuses on the quality of life of patients and families, addressing physical, psychosocial and spiritual concerns through early identification and comprehensive assessment.^10^

KSC applies to all with kidney disease, regardless of CKD grade or the treatment modality, including those who approach kidney failure and are undergoing or considering withdrawal from dialysis and those who have a kidney transplant and may be deteriorating from a failing kidney transplant or increasing comorbidities.^9–11^ KSC is a model of healthcare which is operationalised through multiple interacting components, including interventions for symptom management, shared decision-making, advance care planning, crisis planning, spiritual care, integrated community services, end of life care and bereavement.^10^ KSC could be provided throughout the illness trajectory, at different time points where patients experience critical or worsening clinical conditions and changes in priorities of care.^12^

Though KSC has been recognised as generating better palliative care outcomes, including quality of life,^13^ symptom management^14^ and health services utilisation^15 16^ for certain individuals, some have shown inconclusive findings, particularly with the type and domain of intervention.^17^ Also, diverse study designs of the KSC interventions and varying quality of evidence have potentially limited the generation of definitive conclusions in certain patient and caregiver level outcomes, such as quality of life, symptom management, advance directive completion and patient and caregiver satisfaction due to moderate to high risk of bias.^17^ A recent systematic review found that integrated KSC models and interventions have a positive impact on patient engagement, quality of life, symptom control and health services utilisation; however, further research has been recommended to standardise those interventions to enhance their efficacy and effectiveness, particularly across different populations and socio-cultural and palliative care contexts.^17^

Accordingly, variation in study designs and evidence quality has limited the ability to draw firm conclusions about outcomes of KSC interventions. Existing research also provides little insight into contextual factors such as social, cultural and ethical influences that shape how KSC interventions operate. A realist approach addresses this gap by exploring the mechanisms, or underlying processes and participant responses, that interventions trigger in different contexts to produce outcomes. Realist understanding of this phenomenon will also provide information about the relative effectiveness of components of an intervention, thereby enabling practitioners or service providers to implement effective context-specific interventions under available resources and training.^18^

A recent qualitative evidence synthesis has suggested that ongoing optimisation of policies and frameworks is essential for sustainable adoption of KSC in real-world clinical practice.^19^ However, with the inherent complexity of KSC (with multiple interconnected components, various implementation strategies and multidisciplinary stakeholder involvement) delivered to adults receiving haemodialysis across diverse contexts, including different care setups (eg, hospital: inpatient or outpatient, clinics, care homes, community, etc), and individual circumstances (eg, clinical and health-related factors, socio-economic status, family support, cultural background, educational level, geographical location, etc), often leading to inconsistent or inconclusive outcomes. Therefore, it is unclear from the existing evidence how, for whom and in what circumstances KSC works (or does not work), making it difficult to optimise policies and frameworks relevant to KSC to deliver person-centred care.

In fact, the delivery of KSC is inherently complex and may be dependent on its implementation context, due to the above dynamics; therefore, the rapid realist approach is well-suited to explore how, why, for whom and under what circumstances KSC generates palliative care outcomes rather than asking simply whether KSC works. Despite the potential value of KSC in improving health-related quality of life and holistic well-being of patients and their families, it remains inconsistently implemented and poorly understood within many healthcare systems. Immediate focus on this emerging phenomenon is therefore needed to strengthen evidence and understand influential mechanisms to develop context-specific KSC models. Therefore, it is crucial in exploring the abstract mechanisms that work within and across different contexts and to understand how these produce intended or unintended outcomes.^20^

KSC is underutilised in haemodialysis services^21 22^ and is reported to have inconclusive outcomes due to variability in intervention design, small sample sizes and methodological heterogeneity with available evidence. To date, no studies have attempted to understand the different contexts and mechanisms that influence the outcomes of KSC interventions for adults undergoing haemodialysis, which might be helpful to adjust the implementation context to make it work better. Therefore, this rapid realist review will be timely in building the evidence base to propose policy-relevant recommendations to improve the quality of KSC services for this patient population.

## Methods and analysis

This rapid realist review will be guided by the methodological framework proposed by Saul and colleagues,^23^ consistent with the Realist and Meta-narrative Evidence Syntheses: Evolving Standards (RAMESES) publication standards for realist synthesis.^24^ The review commenced in May 2025 and is anticipated to be completed by May 2026.

### Aim

To explore how KSC interventions work for adults receiving haemodialysis by identifying the contexts in which they are delivered and the mechanisms through which they produce palliative care outcomes.

### Study design

#### Rapid realist review methodology

Realist review is a theory-driven secondary research approach which synthesises diverse evidence from multiple sources, including published literature, policy documents and grey literature^25^ to unpack underlying causation between contexts and mechanisms to generate a particular outcome of an intervention.^26^ A key characteristic of realist inquiry is identifying causal relationships through context–mechanism–outcome configurations, exploring the mechanisms (underlying processes or participant responses) that generate outcomes in specific contexts.^25^ The realist approach is particularly important when exploring a complex phenomenon or similar ‘families’ of social interventions that occur across different contexts and are associated with inconsistent outcomes.^27 28^

‘Rapid realist review’ applies the realist approach to synthesise knowledge on time-sensitive or emerging issues that is particularly useful for policymaking when there is limited time and resources available.^23^ This approach does not typically involve an exhaustive and comprehensive peer-reviewed literature search as it does in the traditional realist review, whereas it involves more purposive and highly focused evidence and may depend on the input from an expert panel or a stakeholder group (knowledge users, patient representatives) to develop programme theories.^23 29^ The time frame is typically shorter for rapid realist reviews (3–6 months turnaround) compared with traditional reviews, which generally take 12–24 months turnaround.^23^ The involvement of diverse expertise groups ensures the validity of the review findings, bringing their on-ground experience on board to understand the emerging mechanisms that occur across various implementation contexts^23 29^ and streamlining the review process to complete within a short period of time.^23 27^ The final output of this review may have potential value for policymakers, service providers and practitioners to improve the quality of KSC delivery in the context of haemodialysis and achieve the desired outcomes through tailored interventions.

### Expert panel

The expert panel for this review comprises palliative medicine consultants, nephrologists (specialised medical practitioners), clinical nurse academics (specialised in nephrology nursing), allied health professionals representing national and international nephrology and palliative care-related advisory boards and patient representatives from a patient and public involvement group. The experts are selected purposively and invited based on their specialty and expertise in the field to inform, refine and validate the developing programme theories. A panel of 10 experts are invited to be involved in three expert panel meetings for theory sensitisation, theory development and refinement and theory validation. In each meeting, the developing programme theories will be presented as if-then-because statements to the experts and their insights will be obtained regarding the real-world applicability of those theories.

Since the experts contributing to this rapid realist review are from various geographical and healthcare contexts, the conflicting perspectives among experts on developing programme theories are treated as valuable indicators explaining how and why KSC interventions work or do not work across different contexts. However, if there are conflicting perspectives among experts on the same programme theories, these will be resolved and prioritised based on the evidence from the literature review.

### Review stages

#### Steps 1 and 2: Defining review scope and development of research questions

**Figure 1:**
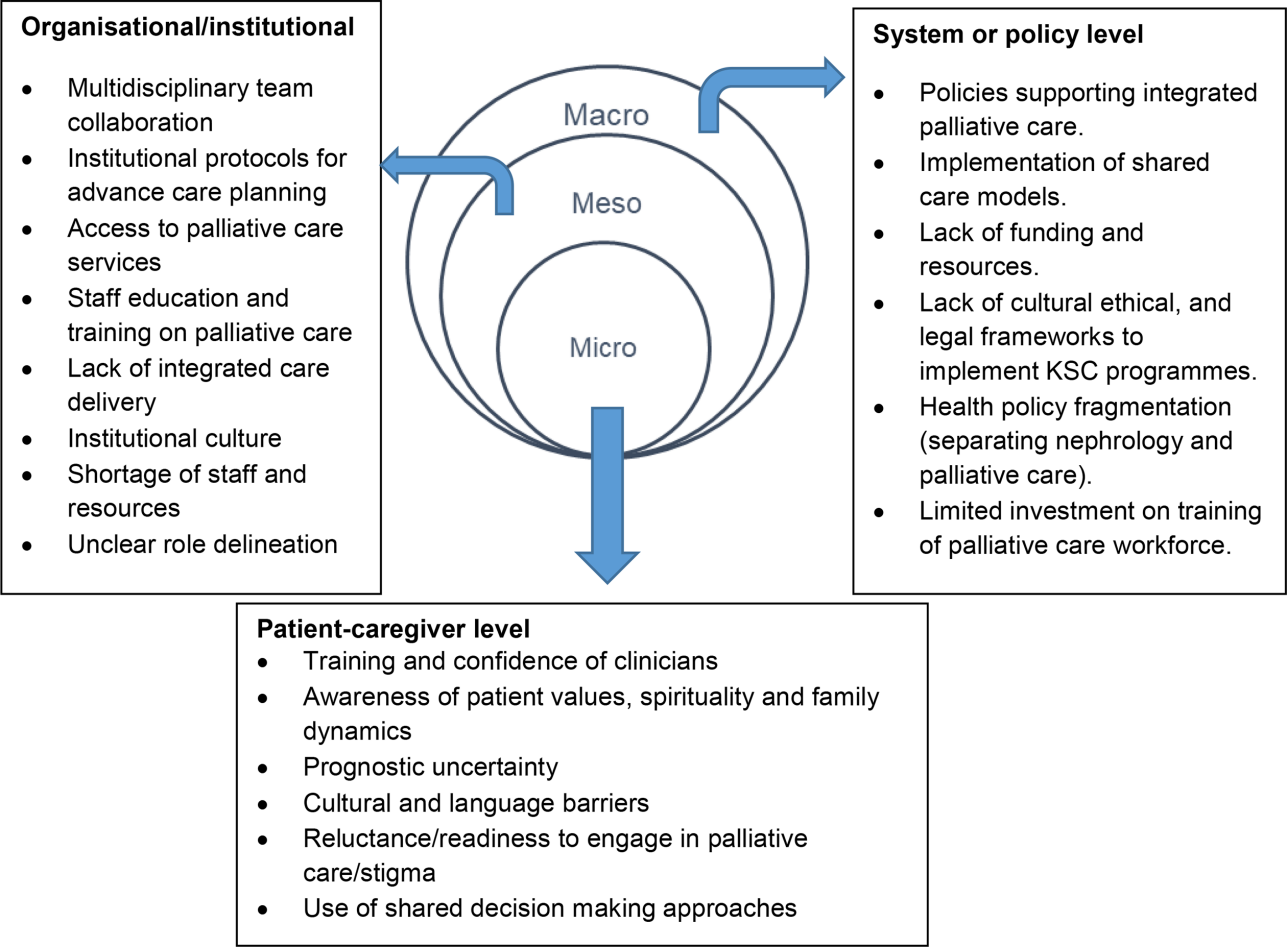
Framework for the influences of implementing kidney supportive care/renal palliative care interventions. KSC, kidney supportive care.

**Figure 2:**
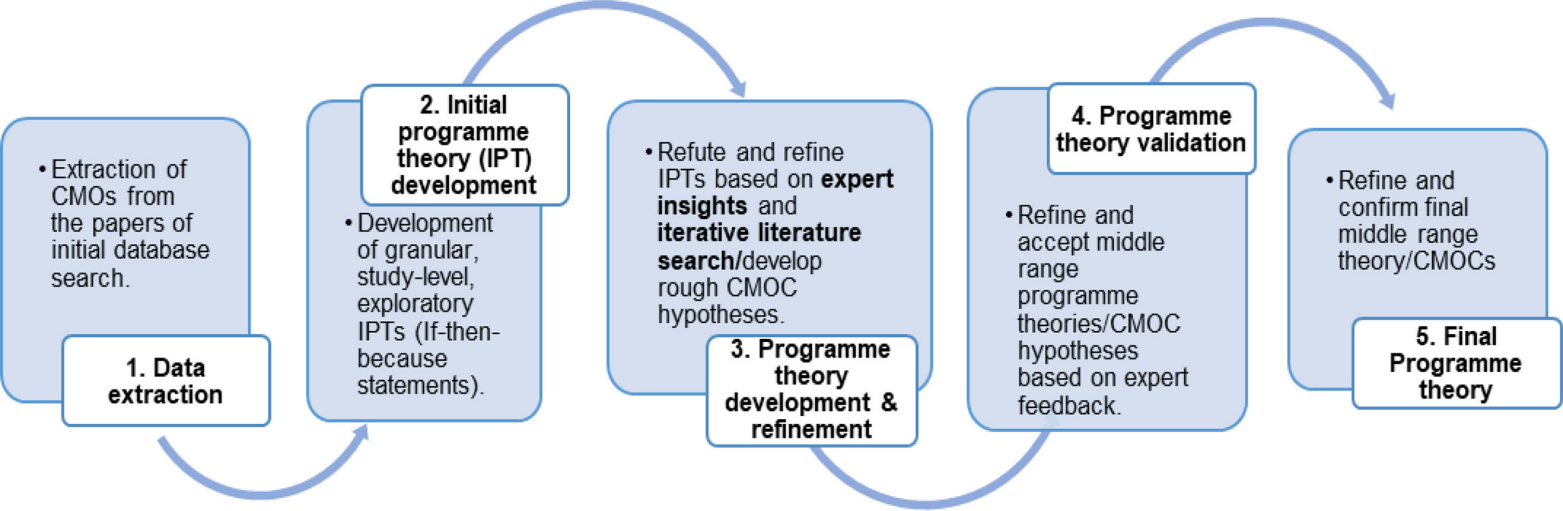
Flow diagram for data extraction and synthesis process. CMOCs, context-mechanism–outcome configurations.

The project scope (content area of interest for the review) was developed with the nephrology and palliative care experts in the supervisory team, who are also actively involved in clinical practice. Further, a preliminary search of existing literature was conducted to identify documents describing KSC interventions and their palliative care outcomes (eg, symptom control, health-related quality of life, functional status, psycho-spiritual well-being, family caregiver burden/strain, advance care planning, patient and caregiver satisfaction, care and death in a preferred location, decisional satisfaction/regret/conflict, good bereavement outcomes, health services utilisation and survival) as well as facilitators or barriers in implementing such interventions for adults receiving haemodialysis. The preliminary search^17 19 30 31^ informed the development of a framework on the influences of implementing KSC interventions which is presented in figure 1. Figure 2 provides an overview of the rapid realist review design (figure figure 2).

The review questions emerged from the preliminary search, and based on the discussions, were:

1. What are the key context-mechanism–outcome configurations (CMOCs) of KSC interventions for adults receiving haemodialysis?
2. How do these CMOCs shape the experiences and outcomes of adults receiving haemodialysis and their family caregivers?
3. How do these CMOCs influence the practices and experiences of healthcare professionals delivering KSC?
4. How do organisational contexts and service structures interact with CMOCs to affect intervention outcomes?

#### Step 3: Identification of how findings and recommendations will be used

A purposive statement was formulated about how to use the findings by the target audience after identifying the review scope and research questions. Accordingly, this rapid realist review aims to understand how, why, for whom and under what circumstances KSC interventions work (or do not work) to generate palliative care outcomes for adults receiving haemodialysis. Using the rapid realist approach, the review will identify CMOCs to better understand the factors that influence the success or failure of these interventions. The findings will inform clinicians, service providers and policymakers to make evidence-informed decisions in the design and delivery of quality KSC service for individuals receiving haemodialysis.

#### Steps 4 and 5: Literature search

The initial search for evidence will be conducted, including a wide range of databases, with the support of an information specialist. The search terms will be developed based on the preliminary searches of the literature and discussions with subject experts in the research team. The key search terms identified are given in table 1. The primary search of literature will be conducted in the databases MEDLINE (PubMed), Cumulative Index of Nursing and Allied Health Literature (CINAHL), Cochrane Central Register of Controlled Trials (CENTRAL), PsycINFO, Scopus and Academic Search Complete using tailored versions of the search strategy formulated using identified search terms in collaboration with a specialist University Librarian. The initial search strategy was formulated to maximise the sensitivity across the spectrum of advanced CKD and palliative or supportive care, ensuring no relevant literature was missed during the initial literature identification stage. While the initial search strategies capture broad disease-specific and dialysis-related studies, the operational scope and iterative literature search of this rapid realist review will strictly focus on haemodialysis-related studies. The full search strategy for the broad initial literature search is given in online supplemental file 1.

**Table 1:** Key search terms

Chronic kidney/renal disease Chronic kidney/renal failure End stage kidney/renal disease End stage kidney/renal failure Advanced kidney/renal disease Advanced kidney/renal failure Chronic kidney/renal impairment Chronic kidney/renal insufficiency Chronic kidney/renal dysfunction Irreversible kidney/renal failure Impaired kidney/renal function dialysis	Palliative care Terminal care End of life care Supportive care Hospice care Comfort care Advance care planning Anticipatory care planning Conservative care Dying

The initial searches will identify literature published from January 2020 to March 2025 to include the most recent evidence, aligning with current operational realities to identify causal insights that are highly dependent on the implementation context, which rapidly changes with time.

All citations of database searches will be exported to reference management software (Rayyan). One reviewer will perform screening of titles and abstracts for relevance based on pre-defined inclusion and exclusion criteria to identify potentially eligible studies to include in the review (table 2). A second reviewer will independently perform the same screening process with 30% of the abstracts following the Cochrane recommendations for rapid review guidance for titles and abstract screening,^32^ to ensure reliability of the screening process and to minimise bias while accelerating and streamlining the review process within the limited time frame.

**Table 2:** Inclusion and exclusion criteria

Inclusion criteria	
Population (P)	**For intervention studies**—studies involving adults over 18 years of age (>18) with a confirmed diagnosis of stage 5 chronic kidney disease (kidney failure) undergoing haemodialysis or dialysis discontinuation. For studies *detailing facilitators, barriers, challenges—in addition to the above criteria for patients, perspectives or experiences on KSC or any co-component of KSC—shared decision-making, symptom management, crisis planning, advance care planning, spiritual care, integration with community services and end of life care and bereavement,^10^ among family caregivers or healthcare professionals caring for the above patients.
Intervention (I)	Studies detailing* KSC interventions (or interventions aligning with any co-component of KSC), particularly those reporting *palliative care outcomes of those interventions. Multicomponent/complex KSC interventions based on a model of palliative care. Studies detailing* facilitators, barriers or challenges in the implementation of KSC interventions.
Comparison (C)	Usual/routine nephrology care/no comparator (pre–post design).
Outcomes (O)	**Validated palliative care outcomes**—symptom control, health-related quality of life, functional status, psycho-spiritual well-being, family caregiver burden/strain, advance care planning, patient and caregiver satisfaction, care and death in a preferred location, decisional satisfaction/regret/conflict, good bereavement outcomes, health services utilisation and survival. **Kidney-specific/clinical outcomes** Discontinuation of dialysis.
Study design (S)	For intervention studies: Only primary studies—involving randomised and non-randomised study types (all forms of study design, including those using qualitative, quantitative, and/or mixed methods approaches). Only qualitative studies were considered for studies detailing facilitators, barriers, or challenges to KSC/renal palliative care.
Exclusion criteria	
Studies involving children (age<18 years). Studies involving adults with AKI. Studies only involving adults in the pre-dialysis stage, peritoneal dialysis, kidney transplant or post-transplant or conservatively managed patients. Studies of supportive care interventions for adults with AKI or children. Studies involving other life-limiting illnesses (multiple long-term conditions), in addition to CKD, undergoing haemodialysis. Studies involving simple/single-component interventions or reporting impact on a single symptom or outcome. Palliative care outcomes reported without evidence of implementing a kidney supportive care/renal palliative care intervention. Letters, opinion pieces or editorials, conference abstracts/proceedings, communications, review papers (review, systematic reviews, narrative review, integrative review, scoping review, etc), debates, perspectives. Studies not published in English.	

* To include a paper in the review, it is necessary to provide detailed information about the intervention that was being used, particularly describing the context in which the intervention has been implemented, and the possible mechanisms involved in generating the reported palliative care outcomes.

AKI, acute kidney injury; CKD, chronic kidney disease; KSC, kidney supportive care.

The full texts of all eligible articles will be retrieved and their relevance checked further by one reviewer. Three reviewers will blindly assess 30% of full-text papers for relevance and to ensure accuracy of the screening process. All excluded papers will be assessed by the research team to ensure no potentially relevant studies are excluded. A data extraction form, designed to record key data, will be used to extract information from short-listed articles. As the searches progress, based on the theory development, the search strategies will be modified iteratively to identify the most relevant documents/articles related to the review question, ensuring that all possible and important documents are included for short-listing in the review. Where appropriate, searches will be further expanded through backward (reference lists of eligible sources) and forward (using the cited by option in Google Scholar and Scopus) citation tracking to identify additional relevant literature to support the developing theories.

#### Step 6: Quality appraisal

Quality assessment of the included studies will follow the RAMESES criteria and publication standards based on whether the reported evidence has sufficient rigour and relevance to inform the CMOC development. As part of the appraisal process, two reviewers will independently evaluate the relevance and richness (whether the articles contain information supporting the programme theory and understanding of context, mechanism and outcomes) as well as the rigour (providing credible and trustworthy evidence) of the selected studies.^24^ Any disagreements will be resolved through discussion with a third reviewer to ensure consistency in the inclusion of papers.

The relevance of a paper will be rated as high, moderate or low,^33 34^ and richness will be assessed based on the following criteria.^34 35^

0=nothing of interest, not focused on design, implementation or use; 1=limited data of interest, likely to appear in other articles; 2=limited data of interest, but quick to extract it and could add weight to findings; 3=some good quality data; 4=much valuable data.

Rigour of each paper will be assessed using the Mixed Methods Appraisal Tool^36^ and categorised as follows.

5***** or 100% quality criteria met

4 **** or 80% quality criteria met

3 *** or 60% quality criteria met

2 ** or 40% quality criteria met

1 * or 20% quality criteria met

To ensure the programme theory is sufficiently plausible and trustworthy, RAMESES quality standards will be followed. Once the programme theory has been generated, it will be reviewed by the expert panel/knowledge users to further ensure the validity of the findings.

#### Step 7: Data extraction and synthesis

Pilot data extractions with 10% of included papers will be carried out independently by two reviewers using a bespoke data extraction template to ensure consensus and accuracy. Extracted data will include general characteristics (author, year and country of publication), title, study aim, design, study population, the type of KSC intervention, information available on the contexts (background and aim of the intervention, the context in which the intervention was implemented- geographical location, age of the participants, care setup), mechanisms and outcomes related to the implementation processes, etc. As given in the realist approach, data extraction will focus on the author’s explanations and discussions about how an intervention was supposed to work or not.^18^

Initially, either manual coding or NVivo software will be used to code the relevant sections or texts related to the concepts of the programme theory from full-text documents, and as the synthesis progresses, they will be developed into CMOCs. The data extraction template will be modified and tailored accordingly as the data extraction progresses. After the initial data extraction is completed, the key findings will be summarised and used to develop initial theories focusing on the interactions between context and mechanisms that influence the palliative care outcomes of KSC interventions for adults receiving haemodialysis. An initial list of CMOCs (initial programme theories) will be developed and discussed with the expert panel (practitioners, patients, caregivers, service providers and policymakers) to ensure their interpretations are relevant to the current practice.

**Figure 3:**
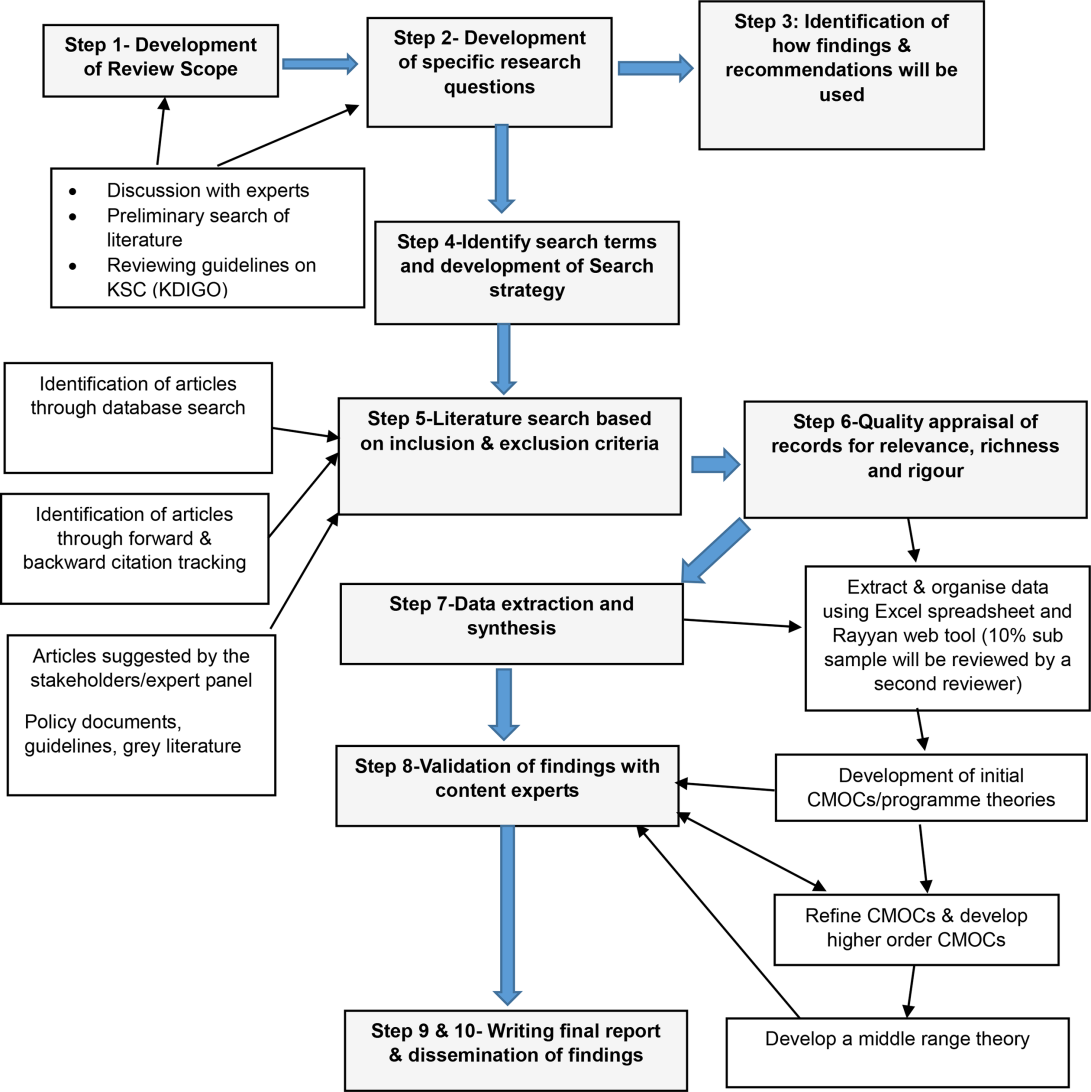
Overview of rapid realist review design. CMOCs, context-mechanism–outcome configurations; KDIGO, Kidney Disease: Improving Global Outcomes; KSC, kidney supportive care.

Following the expert insights, the data extraction will be further carried out with additional documents retrieved through the search process to record the relevant information to formulate or inform the theories described as CMOCs, which form the basis of a realist synthesis. The key emerging findings will be reviewed and discussed in a review team meeting regularly to refine the developing programme theories, ensuring the validity and consistency of the interpretations made. Once the data extraction and theory development have been completed, the key findings will be presented to the expert panel to obtain their feedback to further develop or expand on the findings. Later, in a series of data sessions, these initial theories will be transformed into refined middle-range programme theory with higher-level CMOCs. A final stage will involve the integration of these into a model describing interactions between mechanisms and contexts that influence desired palliative care outcomes of KSC interventions for adults receiving haemodialysis. The data extraction and synthesis process is illustrated in figure 3.

#### Step 8: Validation and refinement

Once the initial programme theories have been formulated, these will be presented to the expert panel who have on-ground experience in the field to ensure the findings apply to real-world clinical practice and address any gaps not covered in the existing literature. Several review team meetings will be held with the purpose of evaluating emerging programme theories in light of the panel’s expertise in providing KSC for adults receiving haemodialysis. The ongoing refinement of programme theories will continue until no additional CMOCs are generated or result in any further modifications to the CMOCs that have already been formulated.^27^ The final output of this rapid realist review will be a refined middle-range theory that can be testable and applicable across multiple contexts, rather than being limited to a single case or setting.^27^

#### Steps 9 and 10: Dissemination of findings

Programme theories will be presented as a means to understand how contextual changes may interact with mechanisms to generate desired outcomes, rather than presenting them as primary findings.^25^ Furthermore, the output of this rapid realist review will include a set of recommendations and a model/conceptual framework, which will be useful in designing context-specific KSC interventions with a more person-centred and holistic approach.

### Strengths and limitations

The strength of this rapid realist review is the involvement of an expert panel throughout the review, which will streamline the process by incorporating insights from their clinical practice and addressing gaps that may exist in the current literature. This process will assist with real-world applicability and produce a comprehensive understanding of what works, for whom and in what circumstances within a short time frame. Since this is a rapid approach, the review will limit exhaustive searching of evidence, which might potentially lead to missing some important information, particularly published before the selected time frame (within the past 6 years) and those available in languages other than English. Therefore, the generalisability of findings may also be limited to some other non-English speaking countries and healthcare systems that might operate with different frameworks and face distinct challenges. Further, this review only focuses on KSC interventions implemented for adults receiving haemodialysis. Therefore, the findings of this review may not be transferable to the other forms of treatment modalities (conservative kidney management, peritoneal dialysis and kidney transplantation) or to a broader CKD management context.

## Ethics and dissemination

Ethics approval for this review was granted through Coventry University Ethics Committee (Ref-P182536). The findings of this review will be disseminated through scientific communications, including peer-reviewed journal publications and conference presentations. Additionally, the review findings and recommendations will be shared with relevant stakeholders, thereby strengthening the evidence base for embedding palliative principles within nephrology, particularly in haemodialysis to address unmet, context-specific palliative care needs in this population. This is crucial for shaping future policies, education and clinical practice in KSC.

## Supplementary material

10.1136/bmjopen-2025-114188 online supplemental file 1
