# Supplementary material for: Understanding the implementation of kidney supportive care interventions to improve palliative care outcomes for adults receiving haemodialysis: protocol for a rapid realist review
Source: BMJ Open. 2026 Jul 21;16(7):e114188. doi: 10.1136/bmjopen-2025-114188 (PMC13405006; doi:10.1136/bmjopen-2025-114188)
Supplement: online supplemental file 1 [file bmjopen-16-7-s001.docx]

**MEDLINE**

**1.**

“Chronic kidney disease*” OR “Chronic renal disease*” OR “Chronic kidney failure*” OR “Chronic renal failure*” OR “End stage kidney disease*” OR“ End stage renal disease*” OR "End stage renal failure*" OR "End stage kidney failure*"OR "Advanced renal disease*" OR "Advanced kidney disease*" OR "Advanced kidney failure*" OR "Advanced renal failure*" OR "Chronic kidney impairment*" OR "Chronic renal impairment*" OR "Chronic kidney insufficienc*" OR "Chronic renal insufficienc*" OR "Chronic kidney dysfunction*" OR "Chronic renal dysfunction*" OR "Irreversible kidney failure*" OR "Irreversible renal failure*" OR “Impaired kidney function*” OR “Impaired renal function*” OR “dialysis” OR (MM "Kidney Failure, Chronic") OR (MM "Renal Insufficiency, Chronic")

**2.**

Palliat* OR Terminal* OR “End of Life” OR Supportive OR Hospice OR “Comfort care” OR “Advance* Care Planning” OR “Anticipatory care planning” OR Conservative* OR Dying OR (MH "Palliative Care") OR (MH "Spiritual Care") OR (MH "Terminal Care") OR (MH "Hospice Care") ) OR ( (MH "Advance Care Planning") OR (MH "Decision Making")

**1 AND 2** (Limiters - Publication Date: 20200101-20250331; English Language; Human; Age Related: All Adult: 19+ years)

**CINAHL**

**1.**

“Chronic kidney disease*” OR “Chronic renal disease*” OR “Chronic kidney failure*” OR “Chronic renal failure*” OR “End stage kidney disease*” OR “End stage renal disease*” OR "End stage renal failure*" OR "End stage kidney failure*"OR "Advanced renal disease*" OR "Advanced kidney disease*" OR "Advanced kidney failure*" OR "Advanced renal failure*" OR "Chronic kidney impairment*" OR "Chronic renal impairment*" OR "Chronic kidney insufficienc*" OR "Chronic renal insufficienc*" OR "Chronic kidney dysfunction*" OR "Chronic renal dysfunction*" OR "Irreversible kidney failure*" OR "Irreversible renal failure*" OR “Impaired kidney function*” OR “Impaired renal function*” OR “dialysis”

**2.**

Palliat* OR Terminal* OR “End of Life” OR Supportive OR Hospice OR “Comfort care” OR “Advance* Care Planning” OR “Anticipatory care planning” OR Conservative* OR Dying OR (MH "Palliative Care") OR (MH "Spiritual Care") OR (MH "Terminal Care") OR (MH "Hospice Care") ) OR ( (MH "Advance Care Planning") OR (MH "Decision Making")

**1 AND 2** (Limiters - Publication Date: 20200101-20250331; English Language; Human; Age Groups: All Adult)

**PsycINFO**

**1.**

“Chronic kidney disease*” OR “Chronic renal disease*” OR “Chronic kidney failure*” OR “Chronic renal failure*” OR “End stage kidney disease*” OR “End stage renal disease*” OR "End stage renal failure*" OR "End stage kidney failure*"OR "Advanced renal disease*" OR "Advanced kidney disease*" OR "Advanced kidney failure*" OR "Advanced renal failure*" OR "Chronic kidney impairment*" OR "Chronic renal impairment*" OR "Chronic kidney insufficienc*" OR "Chronic renal insufficienc*" OR "Chronic kidney dysfunction*" OR "Chronic renal dysfunction*" OR "Irreversible kidney failure*" OR "Irreversible renal failure*" OR “Impaired kidney function*” OR “Impaired renal function*” OR “dialysis”

**2.**

Palliat* OR Terminal* OR “End of Life” OR Supportive OR Hospice OR “Comfort care” OR “Advance* Care Planning” OR “Anticipatory care planning” OR Conservative* OR Dying OR ((DE "Treatment Refusal" OR DE "Palliative Care" OR DE "Terminally Ill Patients" OR DE "Treatment Withholding" OR DE "Hospice" OR DE "Spiritual Care" OR MM "Symptoms Based Treatment" OR MM "Pain Management"

**1 AND 2** (Limiters - Publication Date: 20200101-20250331; English language; Population Group: Human, Male, Transgender, Female, Inpatient, Outpatient)

**Academic Search Complete**

( “Chronic kidney disease*”OR “Chronic renal disease*” OR “Chronic kidney failure*” OR “Chronic renal failure*” OR “End stage kidney disease*” OR“End stage renal disease*” OR "End stage renal failure*" OR "End stage kidney failure*"OR "Advanced renal disease*" OR "Advanced kidney disease*" OR "Advanced kidney failure*" OR "Advanced renal failure*" OR "Chronic kidney impairment*" OR "Chronic renal impairment*" OR "Chronic kidney insufficienc*" OR "Chronic renal insufficienc*" OR "Chronic kidney dysfunction*" OR "Chronic renal dysfunction*" OR "Irreversible kidney failure*" OR "Irreversible renal failure*" OR “Impaired kidney function*” OR “Impaired renal function*” OR “dialysis” ) **AND** ( Palliat* OR Terminal* OR “End of Life” OR Supportive OR Hospice OR “Comfort care” OR “Advance* Care Planning” OR “Anticipatory care planning” OR Conservative* OR Dying ) (Limiters - Publication Date: 20200101-20250331; Publication Type: Academic Journal, Conference Paper, Educational Report, Grey Literature, Review; Document Type: Article, Book Chapter, Book Review, Case Study, Literature Review, Proceedings; Language: English)

**SCOPUS**

( TITLE-ABS-KEY ( "Chronic kidney disease*" OR "Chronic renal disease*" OR "Chronic kidney failure*" OR "Chronic renal failure*" OR "End stage kidney disease*" OR "End stage renal disease*" OR "End stage renal failure*" OR "End stage kidney failure*" OR "Advanced renal disease*" OR "Advanced kidney disease*" OR "Advanced kidney failure*" OR "Advanced renal failure*" OR "Chronic kidney impairment*" OR "Chronic renal impairment*" OR "Chronic kidney insufficienc*" OR "Chronic renal insufficienc*" OR "Chronic kidney dysfunction*" OR "Chronic renal dysfunction*" OR "Irreversible kidney failure*" OR "Irreversible renal failure*" OR "Impaired kidney function*" OR "Impaired renal function*" OR "dialysis" ) **AND** TITLE-ABS-KEY ( palliat* OR terminal* OR "End of Life" OR supportive OR hospice OR "Comfort care" OR "Advance* Care Planning" OR "Anticipatory care planning" OR conservative* OR dying ) ) (AND PUBYEAR > 2019 AND PUBYEAR < 2026 AND ( LIMIT-TO ( SUBJAREA , "MEDI" ) OR LIMIT-TO ( SUBJAREA , "NURS" ) OR LIMIT-TO ( SUBJAREA , "SOCI" ) OR LIMIT-TO ( SUBJAREA , "HEAL" ) OR LIMIT-TO ( SUBJAREA , "ARTS" ) OR LIMIT-TO ( SUBJAREA , "MULT" ) OR LIMIT-TO ( SUBJAREA , "PSYC" ) OR EXCLUDE ( SUBJAREA , "BIOC" ) OR EXCLUDE ( SUBJAREA , "PHAR" ) OR EXCLUDE ( SUBJAREA , "ENVI" ) OR EXCLUDE ( SUBJAREA , "NEUR" ) OR EXCLUDE ( SUBJAREA , "IMMU" ) OR EXCLUDE ( SUBJAREA , "AGRI" ) OR EXCLUDE ( SUBJAREA , "ECON" ) OR EXCLUDE ( SUBJAREA , "MATH" ) OR EXCLUDE ( SUBJAREA , "ENGI" ) OR EXCLUDE ( SUBJAREA , "DENT" ) OR EXCLUDE ( SUBJAREA , "COMP" ) OR EXCLUDE ( SUBJAREA , "CENG" ) ) AND ( EXCLUDE ( DOCTYPE , "ed" ) OR EXCLUDE ( DOCTYPE , "le" ) OR EXCLUDE ( DOCTYPE , "no" ) OR EXCLUDE ( DOCTYPE , "tb" ) OR LIMIT-TO ( DOCTYPE , "ar" ) OR LIMIT-TO ( DOCTYPE , "re" ) OR LIMIT-TO ( DOCTYPE , "cp" ) OR LIMIT-TO ( DOCTYPE , "sh" ) OR LIMIT-TO ( DOCTYPE , "er" ) OR LIMIT-TO ( DOCTYPE , "ch" ) ) AND ( EXCLUDE ( EXACTKEYWORD , "Amino Terminal Pro Brain Natriuretic Peptide" ) OR EXCLUDE ( EXACTKEYWORD , "Creatinine" ) OR EXCLUDE ( EXACTKEYWORD , "Biological Marker" ) OR EXCLUDE ( EXACTKEYWORD , "Creatinine Blood Level" ) OR EXCLUDE ( EXACTKEYWORD , "Blood" ) OR EXCLUDE ( EXACTKEYWORD , "Acute Kidney Failure" ) OR EXCLUDE ( EXACTKEYWORD , "Echocardiography" ) OR EXCLUDE ( EXACTKEYWORD , "Body Mass" ) OR EXCLUDE ( EXACTKEYWORD , "Computer Assisted Tomography" ) OR EXCLUDE ( EXACTKEYWORD , "Heart Left Ventricle Ejection Fraction" ) OR EXCLUDE ( EXACTKEYWORD , "Atrial Fibrillation" ) OR EXCLUDE ( EXACTKEYWORD , "C Reactive Protein" ) OR EXCLUDE ( EXACTKEYWORD , "Biomarkers" ) OR EXCLUDE ( EXACTKEYWORD , "Human Tissue" ) OR EXCLUDE ( EXACTKEYWORD , "Systolic Blood Pressure" ) OR EXCLUDE ( EXACTKEYWORD , "Pathology" ) OR EXCLUDE ( EXACTKEYWORD , "Dipeptidyl Carboxypeptidase Inhibitor" ) OR EXCLUDE ( EXACTKEYWORD , "Brain Natriuretic Peptide" ) OR EXCLUDE ( EXACTKEYWORD , "Angiotensin Receptor Antagonist" ) OR EXCLUDE ( EXACTKEYWORD , "Metabolism" ) OR EXCLUDE ( EXACTKEYWORD , "Child" ) OR EXCLUDE ( EXACTKEYWORD , "Unclassified Drug" ) OR EXCLUDE ( EXACTKEYWORD , "Beta Adrenergic Receptor Blocking Agent" ) OR EXCLUDE ( EXACTKEYWORD , "Proteinuria" ) OR EXCLUDE ( EXACTKEYWORD , "Albumin" ) OR EXCLUDE ( EXACTKEYWORD , "Diagnostic Imaging" ) OR EXCLUDE ( EXACTKEYWORD , "Nonhuman" ) OR EXCLUDE ( EXACTKEYWORD , "Acute Kidney Injury" ) OR EXCLUDE ( EXACTKEYWORD , "Adolescent" ) OR EXCLUDE ( EXACTKEYWORD , "Parathyroid Hormone" ) OR EXCLUDE ( EXACTKEYWORD , "Diastolic Blood Pressure" ) OR EXCLUDE ( EXACTKEYWORD , "Physiology" ) OR EXCLUDE ( EXACTKEYWORD , "Protein Blood Level" ) OR EXCLUDE ( EXACTKEYWORD , "Coronavirus Disease 2019" ) OR EXCLUDE ( EXACTKEYWORD , "Artificial Ventilation" ) OR EXCLUDE ( EXACTKEYWORD , "Calcium" ) OR EXCLUDE ( EXACTKEYWORD , "Nuclear Magnetic Resonance Imaging" ) OR EXCLUDE ( EXACTKEYWORD , "Obesity" ) OR EXCLUDE ( EXACTKEYWORD , "Kidney Biopsy" ) OR EXCLUDE ( EXACTKEYWORD , "Diuretic Agent" ) OR EXCLUDE ( EXACTKEYWORD , "Natriuretic Peptide, Brain" ) OR EXCLUDE ( EXACTKEYWORD , "Peptide Fragment" ) OR EXCLUDE ( EXACTKEYWORD , "Peptide Fragments" ) OR LIMIT-TO ( EXACTKEYWORD , "Human" ) OR LIMIT-TO ( EXACTKEYWORD , "Humans" ) ) AND ( LIMIT-TO ( LANGUAGE , "English" ) )

**Cochrane Library (CENTRAL)**

**1.**

"Chronic kidney disease*" OR "Chronic renal disease*" OR "Chronic kidney failure*" OR "Chronic renal failure*" OR "End stage kidney disease*" OR "End stage renal disease*" OR "End stage renal failure*" OR "End stage kidney failure*" OR "Advanced renal disease*" OR "Advanced kidney disease*" OR "Advanced kidney failure*" OR "Advanced renal failure*" OR "Chronic kidney impairment*" OR "Chronic renal impairment*" OR "Chronic kidney insufficienc*" OR "Chronic renal insufficienc*" OR "Chronic kidney dysfunction*" OR "Chronic renal dysfunction*" OR "Irreversible kidney failure*" OR "Irreversible renal failure*" OR "Impaired kidney function*" OR "Impaired renal function*" OR "dialysis"):ti,ab,kw

**2.**

MeSH descriptor: [Kidney Failure, Chronic]

**3.**

(Palliat* OR Terminal* OR “End of Life” OR Supportive OR Hospice OR “Comfort care” OR “Advance* Care Planning” OR “Anticipatory care planning” OR Conservative* OR Dying):ti,ab,kw

**4.**

MeSH descriptor: [Palliative Care]

**(#1 OR #2) AND (#3 OR #4)**
