# Supplementary material for: Understanding the implementation of kidney supportive care interventions to improve palliative care outcomes for adults receiving haemodialysis: protocol for a rapid realist review
Source: BMJ Open. 2026 Jul 21;16(7):e114188. doi: 10.1136/bmjopen-2025-114188 (PMC13405006; doi:10.1136/bmjopen-2025-114188)
Supplement: Supplementary file 2 [file bmjopen-2025-114188.reviewer_comments.pdf]

PEER REVIEW HISTORY

BMJ Open publishes all reviews undertaken for accepted manuscripts. Reviewers are asked to complete a checklist review form and are provided with free text boxes to elaborate on their assessment. These free text comments are reproduced below.

ARTICLE DETAILS

Title (Provisional)

Understanding the implementation of kidney supportive care interventions to improve palliative care outcomes for adults receiving haemodialysis: Protocol for a rapid realist review

Authors

Lasanthika, Chalani; Hole, Barnaby; Szczepura, Ala; Bonner, Ann; Coad, Jane; Murphy, Emma

---

VERSION 1 - REVIEW

**Reviewer**	**1**
**Name**	**Li, Guisen**
**Affiliation**	**Institution of Nephrology, Renal Department**
**Date**	**19-Jan-2026**
**COI**	**None**

This protocol presents a well-structured and timely proposal for a rapid realist review aimed at understanding the contextual factors and mechanisms influencing the implementation of Kidney Supportive Care (KSC) interventions for adults on hemodialysis. The study tries to address a gap in the literature and employs an appropriate rapid realist review for exploring complex interventions in varying contexts.

1. The abstract clearly states the aim but could benefit from a more explicit mention of the intended output. Consider adding a sentence summarizing the expected contribution to policy or practice.

2. The introduction effectively outlines the burden of CKD and the need for KSC. However, it would be strengthened by briefly citing recent systematic reviews or meta-analyses that highlight the inconsistent outcomes of KSC interventions, thereby reinforcing the necessity of a realist approach.

3. While databases and timeframe are specified, the protocol should justify the choice of this recent timeframe. A brief rationale for excluding earlier literature would enhance methodological transparency.

4. Table 2 outlines inclusion/exclusion criteria, but the term “detailing” is vague. Please define what level of detail is required to ensure consistent study selection.

5. The expert panel is described but their specific roles in theory development, validation, and interpretation could be elaborated. How will conflicts in perspectives be managed?

6. The description of data extraction and synthesis is appropriate but would benefit from a clearer outline of how CMOCs will be developed iteratively. Consider including a flowchart or stepwise diagram to illustrate the process from data extraction to theory refinement.

7. The process for validating CMOCs with the expert panel is described briefly. Expand on how consensus will be reached, how contradictory interpretations will be handled, and whether a modified Delphi or similar structured approach will be used.

**Reviewer**	**2**
**Name**	**Flynn, Rachel**
**Affiliation**	**University College Cork**
**Date**	**08-Apr-2026**
**COI**	**None**

Overall, this protocol demonstrates a strong understanding of realist methodology and will make an importance contribution to this field. To strengthen the manuscript, I suggest explicitly framing Kidney Supportive Care (KSC) as a complex intervention, clarifying contexts, mechanisms, and outcomes throughout, and improving the linkage between evidence limitations and the rationale for a realist approach. It would also be helpful to illustrate how this review is ‘rapid’ in nature, provide concrete details on the expert panel, palliative care outcomes, and CMOCs, and refine clarity, readability, and consistency across the abstract, background, and methods. Implementing these changes will enhance methodological transparency and align the protocol with BMJ Open reporting expectations for realist review protocols.

Reviewer Feedback: Realist Review Protocol – Kidney Supportive Care (KSC)

Abstract

1. **Clarify KSC as an intervention:** Suggest explicitly stating that Kidney Supportive Care (KSC) is a model of care or intervention. For example (lines 11–15):
   “Chronic Kidney Disease (CKD) is a significant global health concern, contributing substantially to mortality; in advanced stages, patients often require Kidney Supportive Care (KSC), a model of care/intervention that addresses complex palliative and supportive needs.”

2. **Address unmet needs and realist rationale:** Lines 16–20 could be strengthened to reflect realist thinking:

"These needs often remain insufficiently addressed, particularly among patients receiving haemodialysis, due to the interplay of multiple contextual factors and inconsistent reported outcomes. Consequently, it remains unclear how, for whom, and under what circumstances KSC works (or does not work)."

3. **Methods description:** Lines 27–30 should better reflect what realist approaches do: *"Rapid realist review is a structured, theory-driven approach to evidence synthesis conducted within a condensed timeframe, aiming to explain how and why interventions work, for whom, and in what contexts, to identify effective models of service delivery."*
4. **CMOCs and programme theory:** Line 51 should read: *"Data extraction and synthesis will be conducted iteratively to identify context–mechanism–outcome configurations (CMOCs), which will inform the development and refinement of the programme theory, rather than simply informing palliative care outcomes."*
5. **Expert panel:** State the composition of the expert panel, e.g., "An expert panel of [number] will review, evaluate, and reflect on the initial CMOCs in light of their ? clinical expertise and personal experience."
6. **Ethics and dissemination:** Clarify why ethics approval is needed for a review. Is this because of a linked PPI component, or part of a larger study?

Strengths and Limitations

- Emphasise that realist reviews identify **both what works and what does not work**, not only effective interventions.
- Suggest including a simple example of a CMO in this field. For instance: *"A detailed understanding of the mechanisms—defined as the underlying processes, such as participants' responses to resources or changes in care—that lead to successful KSC interventions, and the contexts in which these mechanisms are triggered, will support service redesign."*

Background / Introduction

1. Page 5, line 12: Clearly define the **complexity of care and needs**. Give examples (clinical, psychosocial, spiritual).
2. Page 5 Line 20: Move the definition of KSC (line 29) here to illustrate its complexity: *"The complexity of care of these individuals is often accompanied by numerous supportive care needs requiring palliative care support, referred to as 'renal palliative care' or Kidney Supportive Care (KSC). KSC focuses on the quality of life of patients*

and families, addressing physical, psychosocial, and spiritual concerns through early identification and comprehensive assessment."

3. Provide **key attributes/components of KSC** as an intervention.
4. Clarify what is meant by "certain outcomes" (patient, provider, or system level), give examples of palliative care outcomes.
5. Page 5 Lines 44–51: Improve linkage between diverse study designs and realist rationale. Suggested rewording:
   "Variation in study designs and evidence quality has limited the ability to draw firm conclusions about outcomes. Existing research also provides little insight into contextual factors—such as social, cultural, and ethical influences—that shape how KSC interventions operate. A realist approach addresses this gap by exploring the mechanisms, or underlying processes and participant responses, that interventions trigger in different contexts to produce outcomes."
6. Highlight **inherent complexity** of KSC interventions: multiple interacting components, multidisciplinary involvement, diverse settings (hospital, outpatient, care homes, community), and individual circumstances (clinical, socio-economic, cultural).

Aim

- The aim should explicitly reference **contexts, mechanisms, outcomes, and the target population**.
- Suggested formulation:
  "This rapid realist review aims to explore how Kidney Supportive Care (KSC) interventions work for adults receiving haemodialysis by identifying the contexts in which they are delivered and the mechanisms through which they produce palliative care outcomes."

Methodology

1. Page 7, Line 40: The statement "Realist review is an alternative to systematic reviews that applies realist philosophy to integrate findings from primary studies" is misleading. Realist reviews:
   - Are not limited to primary studies
   - Are more than an alternative to systematic reviews
   - Synthesise diverse evidence to explain how, why, for whom, and under what circumstances interventions produce outcomes
2. Clarify **causal relationships in realist inquiry**:
   - Avoid "underlying mechanisms that link two events"

    - Emphasise **CMO configurations**: mechanisms are processes, reasoning, or participant responses triggered by resources in context
    - Suggested phrasing:
      "A key characteristic of realist inquiry is identifying causal relationships through context–mechanism–outcome configurations, exploring the mechanisms (underlying processes or participant responses) that generate outcomes in specific contexts."

3. Remove repetitive paragraphs (paragraph 1 & 2 are repetitive under Rapid realist review methodology); keep the clearer explanation of realist methodology (paragraph 2).
4. Line 21-38: Description of KSC as complex intervention, this is great but this needs to be presented earlier in the background after defining KSC to illustrate it as a **complex intervention**.
5. Expert panel: Clarify number, selection process, and how CMOCs will be presented (CMO format, if/then statements). Explain how differences in perspectives will be managed.
6. Provide examples of **palliative care outcomes**.
7. Page 8, Line 41: Screening process: Provide rationale for using a second reviewer on 30% of abstracts; typically, two independent reviewers are recommended.
8. Rapid realist review considerations: Clearly describe how this differs from a full realist review, including the timeline.

Research Questions
Suggested framework:

- What are the key CMOCs of KSC interventions for adults receiving haemodialysis?
- How do these CMOCs shape the experiences and outcomes of patients and their family caregivers?
- How do these CMOCs influence the practices and experiences of healthcare professionals delivering KSC?
- How do organisational contexts and service structures interact with CMOCs to affect intervention outcomes?

Summary
Overall, the protocol demonstrates a strong understanding of realist methodology. The feedback focuses on:

- Explicitly framing KSC as a complex intervention
- Clarifying contexts, mechanisms, and outcomes throughout
- Improving linkage between evidence limitations and the rationale for a realist approach
- Illustrate how this is ‘rapid’ in nature
- Providing concrete details on expert panel, palliative care outcomes, and CMOCs
- Refining clarity, readability, and consistency across abstract, background, and methods

Implementing these changes will strengthen the methodological clarity and alignment with BMJ Open reporting expectations for realist review protocols.

VERSION 1 - AUTHOR RESPONSE

Reviewer 1

No	Comment from Reviewer	Response from Authors	Where the changes have been made
1.	The abstract clearly states the aim but could benefit from a more explicit mention of the intended output. Consider adding a sentence summarizing the expected contribution to policy or practice.	Thank you very much for this important comment. We have included the following section describing the impact of this review for policy initiatives, education and clinical practice related to kidney supportive care (KSC) in the abstract.<br><br>*“This rapid realist review will support service redesign to better address the unmet palliative care needs of adults receiving haemodialysis, and inform policy initiatives and education to deliver context-specific KSC across the UK and globally”.*	Abstract<br>Page No. 3<br>(Line Numbers 4-6)
2.	The introduction effectively outlines the burden of CKD and the need for KSC. However, it would be strengthened by	Thank you for raising this valuable comment to include recent systematic review	Introduction<br>Page No.5<br>(Line numbers 2-6 and 16-17)

	briefly citing recent systematic reviews or meta-analyses that highlight the inconsistent outcomes of KSC interventions, thereby reinforcing the necessity of a realist approach.	evidence to reinforce the necessity of a realist approach.<br><br>Accordingly, we have incorporated evidence from systematic reviews in the introduction as follows.<br><br>*"A recent systematic review found that integrated KSC models and interventions have a positive impact on patient engagement, quality of life, symptom control and health services utilisation; however, further research has been recommended to standardise those interventions to enhance their efficacy and effectiveness, particularly across different populations and socio-cultural and palliative care contexts"*<br><br>*"A recent qualitative evidence synthesis has suggested that ongoing optimisation of policies and frameworks is essential for sustainable adoption of KSC in real-world clinical practice".*	
3.	While databases and timeframe are specified, the protocol should justify the choice of this recent timeframe. A brief rationale for excluding earlier literature would enhance methodological transparency.	Thank you for your comment.<br><br>We have included a justification for why we included literature from a short time frame for initial programme theory development, under the subheading 'Literature search' as follows.<br><br>*"The initial searches will identify literature published from January 2020 to March 2025 to*	Methods and analysis- Page No.9 (Line numbers 23-26)

		include the most recent evidence, aligning with current operational realities to identify causal insights that are highly dependent on the implementation context, which rapidly changes with time".	
4.	Table 2 outlines inclusion/exclusion criteria, but the term "detailing" is vague. Please define what level of detail is required to ensure consistent study selection.	Thank you very much for your insightful comment.<br><br>We have provided an explanation of the word 'detailing' at the end of the table as a footnote as follows.<br><br>*"**detailing* -** To include a paper in the review, it is necessary to provide detailed information about the intervention that was being used, particularly describing the context in which the intervention has been implemented, and the possible mechanisms involved in generating the reported palliative care outcomes".*	Footnote-Table 2 (inclusion & exclusion criteria)
5.	The expert panel is described but their specific roles in theory development, validation, and interpretation could be elaborated. How will conflicts in perspectives be managed?	Given that the expert panel comprises representatives from diverse care contexts (from different geographical locations- e.g. England, Scotland, Ireland, Australia and different expertise), their differing perspectives are treated as valuable indicators of how and why the intervention work/does not work across different contexts. They are simply different contexts yielding different outcomes.	Methods and analysis-Page No. 7 (line numbers 19-32) and 8 (Line numbers 1-3)

		Consequently, conflicting perspectives of the panel will only require formal resolution if they present direct contradictions within the same programme theory under identical contextual conditions. However, this is extremely rare in this rapid realist review as experts are coming from different contexts. In any case, the conflicts will be resolved based on the evidence from the literature (prioritising the theory with evidence base).<br><br>We have included a section under the subheading 'expert panel' describing the specific roles of the expert panel and how conflicting perspectives will be managed.	
6.	The description of data extraction and synthesis is appropriate but would benefit from a clearer outline of how CMOCs will be developed iteratively. Consider including a flowchart or stepwise diagram to illustrate the process from data extraction to theory refinement.	Thank you for your suggestion.<br><br>We have included a stepwise diagram to illustrate the process of how CMOCs will be developed, from data extraction to theory refinement with expert insights and iterative literature search.	Figure 3
7.	The process for validating CMOCs with the expert panel is described briefly. Expand on how consensus will be reached, how contradictory interpretations will be handled, and whether a modified Delphi or similar structured approach will be used.	Thank you very much for your thoughtful comment.<br>In realist reviews, conflicting perspectives are treated as natural variations of the implementation context and are treated as valuable indicators generating causal insights about how and why an intervention works or does not work.	Methods and analysis- Page No. 7 (Line numbers 19-32) and 8 (Line numbers 1-3)

		Consequently, conflicting perspectives of the panel will only require formal resolution if they present direct contradictions within the same programme theory under identical contextual conditions. However, this is extremely rare in this rapid realist review as experts are coming from different contexts. In any case, the conflicts will be resolved based on the evidence from the literature (prioritising the theory with evidence base).<br><br>We have included a section under the subheading 'expert panel' describing how conflicting perspectives/ contradictory interpretations will be managed.	

Reviewer 2

No	Comment from Reviewer	Response from Authors	Where the changes have been made
	**Abstract**		
1.	Clarify KSC as an intervention: Suggest explicitly stating that Kidney Supportive Care (KSC) is a model of care or intervention.<br>For example (lines 11–15):<br>*"Chronic Kidney Disease (CKD) is a significant global health concern, contributing substantially to mortality; in advanced stages, patients often require Kidney Supportive Care (KSC), a model of care/intervention that*	Thank you very much for your insightful comment and suggestion to rephrase the sentence.<br><br>We have revised the text as suggested in the abstract.	Abstract<br>Page No.2<br>(Line numbers 5-8)

	addresses complex palliative and supportive needs."		
2.	Address unmet needs and realist rationale: Lines 16–20 could be strengthened to reflect realist thinking: *"These needs often remain insufficiently addressed, particularly among patients receiving haemodialysis, due to the interplay of multiple contextual factors and inconsistent reported outcomes. Consequently, it remains unclear how, for whom, and under what circumstances KSC works (or does not work)."*	Thank you for your insightful suggestion.<br><br>We have revised the text as suggested in the abstract.	Abstract<br>Page No.2<br>(Line numbers 8-11)
3.	Methods description: Lines 27–30 should better reflect what realist approaches do *"Rapid realist review is a structured, theory-driven approach to evidence synthesis conducted within a condensed timeframe, aiming to explain how and why interventions work, for whom, and in what contexts, to identify effective models of service delivery."*	Thank you for your insightful suggestion.<br><br>We have revised the text as suggested in the abstract.	Abstract<br>Page No.2<br>(Line numbers 15-18)
4.	CMOCs and programme theory: Line 51 should read: *"Data extraction and synthesis will be conducted iteratively to identify context mechanism–outcome configurations (CMOCs), which will inform the development and refinement of the programme theory, rather than simply informing palliative care outcomes."*	Thank you for this valuable suggestion.<br><br>We have revised the text in the abstract as suggested.	Abstract<br>Page No.2<br>(line numbers 29-32)
5.	Expert panel: State the composition of the expert	Thank you very much for this important comment.	Abstract

	panel, e.g., "An expert panel of [number] will review, evaluate, and reflect on the initial CMOCs in light of their ? clinical expertise and personal experience."	We have included the following sentence in the abstract to include the composition of the expert panel.<br>*"A panel of 10 experts will review, evaluate, and reflect on the initial CMOCs in light of their clinical expertise and personal experience".*	Page No.3 (Line numbers 1-2)
6.	Ethics and dissemination: Clarify why ethics approval is needed for a review. Is this because of a linked PPI component, or part of a larger study?	Thank you for this comment on why ethics approval is needed for a review.<br>This review is a part of a PhD study. Ethics approval is a requirement at Coventry University (where the student is registered for a PhD) in all phases of the PhD studies, including systematic reviews. Further, as this study involves an expert panel in addition to the secondary literature search, we ensured that institutional ethics approval is in place prior to conducting the review.	
	**Strengths and Limitations**		
7.	Emphasise that realist reviews identify both what works and what does not work, not only effective interventions.	Thank you for this comment.<br>We have revised the strengths and limitations section, including the following sentence.<br>*"The rapid realist approach is better suited than other empirical methods to understand the mechanisms behind why and how KSC works or does not work for adults receiving haemodialysis, rather than assuming there is one model to suit all situations".*	Strengths and limitations Page No. 3 (Line numbers 14-16)
8.	Suggest including a simple example of a CMO in this	Thank you for your suggestion.	Strengths and limitations

	field. For instance: *"A detailed understanding of the mechanisms—defined as the underlying processes, such as participants' responses to resources or changes in care—that lead to successful KSC interventions, and the contexts in which these mechanisms are triggered, will support service redesign."*	We have revised the strengths and limitations section to include the suggested text.	Page No. 3 (Line numbers 21-24)
	**Background/Introduction**		
9.	Background / Introduction 1. Page 5, line 12: Clearly define the complexity of care and needs. Give examples (clinical, psychosocial, spiritual).	Thank you very much for your comment. We have included the following sentence to define the complexity of care needs.<br><br>*"The care for adults with kidney failure may have to address complex needs, particularly in the clinical, psychosocial, and spiritual domains and require vigilant and respectful coordination between specialised health services, including routine nephrology care, palliative care, and general practice"*	Introduction Page No.4 (Line numbers 10-13)
10.	Page 5 Line 20: Move the definition of KSC (line 29) here to illustrate its complexity: *The complexity of care of these individuals is often accompanied by numerous supportive care needs requiring palliative care support, referred to as 'renal palliative care' or Kidney Supportive Care (KSC). KSC focuses on the quality of life of patients and families, addressing physical, psychosocial, and spiritual*	Thank you for your suggestion.<br><br>The definition of KSC is moved as suggested to illustrate its complexity, following the description of the requirements of palliative care support to address complex needs.	Introduction Page No.4 (Line numbers 13-17)

	concerns through early identification and comprehensive assessment."		
11.	Provide key attributes/components of KSC as an intervention.	Thank you for this insight. We have included a sentence describing key attributes/components of KSC as an intervention as follows.<br>*"KSC is a model of healthcare which is operationalised through multiple interacting components, including interventions for symptom management, shared decision-making, advance care planning, crisis planning, spiritual care, integrated community services, end of life care and bereavement".*	Introduction Page No.4 (Line numbers 21-24)
12.	Clarify what is meant by "certain outcomes" (patient, provider, or system level), give examples of palliative care outcomes.	Thank you for the comment. We have given examples for palliative care outcomes in the text as given below.<br><br>*"Also, diverse study designs of the KSC interventions and varying quality of evidence have potentially limited the generation of definitive conclusions in certain patient and caregiver level outcomes, such as quality of life, symptom management, advance directive completion, and patient and caregiver satisfaction due to moderate to high risk of bias".*	Introduction Page No.4 (Line numbers 30-32) and 5 (Line numbers 1-2)
13.	Page 5 Lines 44–51: Improve linkage between diverse study designs and realist rationale. Suggested rewording: *"Variation in study designs and evidence quality has limited the ability to draw firm*	Thank you for this insightful suggestion.<br><br>We have rephrased the text as suggested.	Introduction Page No.5 (Line numbers 7-12)

	conclusions about outcomes. Existing research also provides little insight into contextual factors—such as social, cultural, and ethical influences—that shape how KSC interventions operate. A realist approach addresses this gap by exploring the mechanisms, or underlying processes and participant responses, that interventions trigger in different contexts to produce outcomes."		
14.	Highlight inherent complexity of KSC interventions: multiple interacting components, multidisciplinary involvement, diverse settings (hospital, outpatient, care homes, community), and individual circumstances (clinical, socio-economic, cultural).	Thank you very much for this comment.<br><br>We have included a text highlighting why KSC is inherently complex in the introduction as follows.<br><br>*"However, with the inherent complexity of KSC (with multiple interacting components, various implementation strategies and multidisciplinary stakeholder involvement) delivered to individuals receiving haemodialysis across diverse contexts, including different care setups (e.g. hospital: inpatient or outpatient, clinics, care homes, community, etc.), and individual circumstances (e.g. clinical and health-related factors, socio-economic status, family support, cultural background, educational level, geographical location, etc.), often leading to inconsistent or inconclusive outcomes. Therefore, it is unclear from the existing*	Introduction<br><br>Page No.5 (Line numbers 18-26)

		evidence how, for whom, and in what circumstances KSC works (or does not work), making it difficult to optimise policies and frameworks relevant to KSC to deliver person-centred care".	
	**Aim**		
15.	The aim should explicitly reference contexts, mechanisms, outcomes, and the target population.<br>Suggested formulation: *"This rapid realist review aims to explore how Kidney Supportive Care (KSC) interventions work for adults receiving haemodialysis by identifying the contexts in which they are delivered and the mechanisms through which they produce palliative care outcomes."*	Thank you very much for this thoughtful suggestion.<br><br>We have revised the aim of the review to explicitly include contexts, mechanisms, outcomes and the target population as suggested.	Methods and analysis<br>Page No.6<br>(Line numbers 19-21)
	**Methodology**		
16.	Page 7, Line 40: The statement "Realist review is an alternative to systematic reviews that applies realist philosophy to integrate findings from primary studies" is misleading. Realist reviews:<br>o Are not limited to primary studies<br>o Are more than an alternative to systematic reviews o Synthesise diverse evidence to explain how, why, for whom, and under what circumstances interventions produce outcomes	Thank you very much for this important comment about misleading information.<br><br>We have now revised the text to clearly explain what a realist review is, what it involves and its purpose as follows.<br><br>*"Realist review is a 'theory-driven secondary research approach which synthesises diverse evidence from multiple sources, including published literature, policy documents, and grey literature*[25] *to unpack underlying causation between contexts and mechanisms*	Methods and analysis<br>Page No.6<br>(Line numbers 24-27)

		to generate a particular outcome of an intervention[26]".	
17.	Clarify causal relationships in realist inquiry:<br>o Avoid "underlying mechanisms that link two events" o Emphasise CMO configurations: mechanisms are processes, reasoning, or participant responses triggered by resources in context o Suggested phrasing:<br>*"A key characteristic of realist inquiry is identifying causal relationships through context–mechanism–outcome configurations, exploring the mechanisms (underlying processes or participant responses) that generate outcomes in specific contexts."*	Thank you for this important suggestion.<br><br>We have revised the text as suggested to emphasise the formulation of CMO configurations as a key characteristic of realistic inquiry.	Methods and analysis<br>Page No.6 (Line number 27- 30).
18.	Remove repetitive paragraphs (paragraph 1 & 2 are repetitive under Rapid realist review methodology); keep the clearer explanation of realist methodology (paragraph 2).	Thank you for the comment about the repetition of information. We have removed paragraph 1 and kept paragraph 2, which provides a clearer explanation of realist methodology.	Methods and analysis<br>Page No.6 (Line numbers 24-30) and 7 (Line numbers 1-2).
19.	Line 21-38: Description of KSC as complex intervention, this is great but this needs to be presented earlier in the background after defining KSC to illustrate it as a complex intervention.	Thank you very much for this feedback and suggestion. We have moved the relevant text slightly earlier in the introduction to illustrate the complexity of KSC as an intervention.	Introduction<br><br>Page No.5 (Line numbers 18-26)
20.	Expert panel: Clarify number, selection process, and how CMOCs will be presented (CMO format, if/then statements). Explain how	Thank you for this comment. We have provided a description about the expert panel and their role (e.g. number, selection process and how CMOCs will be presented to the experts) and how to manage	Methods and analysis<br>Page No 7 (Line numbers 19-32) and 8

	differences in perspectives will be managed.	conflicting perspectives in the methods and analysis section, under the subheading 'expert panel'.	(Line numbers 1-3)
21.	Provide examples of palliative care outcomes.	Thank you for this comment. We have included examples for palliative care outcomes in the methods and analysis section, under the subheading 'Review stages- Steps 1 and 2: Defining review scope & development of research questions'	Methods and analysis<br>Page No.8 (Line numbers 9-13)
22.	Page 8, Line 41: Screening process: Provide rationale for using a second reviewer on 30% of abstracts; typically, two independent reviewers are recommended.	Thank you for raising this important point.<br>We followed Cochrane recommendations for rapid review guidance for titles and abstract screening, considering the limited time frame to complete the review. The rapid review guidance indicates the following.<br><br>"Use two reviewers for dual screen of at least 20% (ideally more) of abstracts, with conflict resolution" (Garritty C, Gartlehner G, Kamel C, King VJ, Nussbaumer-Streit B, Stevens A, Hamel C, Affengruber L. Cochrane Rapid Reviews. Interim Guidance from the Cochrane Rapid Reviews Methods Group. March 2020)<br><br>Accordingly, we have included a sentence explaining the rationale for the use 30% screening of abstracts by a second reviewer as follows.<br><br>*"A second reviewer will independently perform the same*	Methods and analysis<br><br>Page No. 9(Line numbers 30-32) and 10 (Line numbers 1-2)

		screening process with 30% of the abstracts following the Cochrane recommendations for rapid review guidance for titles and abstract screening, to ensure reliability of the screening process and to minimise bias, accelerating and streamlining the review process within the limited time frame".	
23.	Rapid realist review considerations: Clearly describe how this differs from a full realist review, including the timeline.	Thank you for this comment to provide the difference between a rapid realist review and a full realist review.<br><br>We have included a text in the methods and analysis section to explain why the rapid realist review was considered and how it differs from a full realist review, as follows.<br><br>*"This approach does not typically involve an exhaustive and comprehensive peer-reviewed literature search as it does in the traditional realist review, whereas it involves more purposive and highly focused evidence and may depend on the input from an expert panel or a stakeholder group (knowledge users, patient representatives) to develop programme theories[23,29]. The time frame is typically shorter for rapid realist reviews (3-6 months turnaround) compared to traditional reviews, which generally take 12-24 months turnaround[23]"*	Methods and analysis Page No.7 (Line numbers 5-11)
	**Research Questions**		

24.	Suggested framework:<br>What are the key CMOCs of KSC interventions for adults receiving haemodialysis?<br>• How do these CMOCs shape the experiences and outcomes of patients and their family caregivers? Y<br>• How do these CMOCs influence the practices and experiences of healthcare professionals delivering KSC?<br>• How do organisational contexts and service structures interact with CMOCs to affect intervention outcomes?	Thank you very much for this important suggestion to rephrase the review questions.<br><br>We have revised the review questions as suggested.	Methods and analysis Review stages- Page No.8 (Line numbers 19-25)
